# Health symptom trajectories and neurotoxicant exposures in Gulf War veterans: the Ft. Devens cohort

**DOI:** 10.1186/s12940-021-00812-0

**Published:** 2022-01-08

**Authors:** Maxine H. Krengel, Clara G. Zundel, Timothy Heeren, Megan Yee, Avron Spiro, Susan P. Proctor, Claudia M. Grasso, Kimberly Sullivan

**Affiliations:** 1 Research Service, VA Boston Healthcare System, 150 South Huntington Ave. 8A-90, Jamaica Plain, Boston, MA 02130 USA; 2 Behavioral Neuroscience Program, Boston University School of Medicine, Boston, MA USA; 3 Department of Biostatistics, Boston University School of Public Health, Boston, MA USA; 4 Department of Environmental Health, Boston University School of Public Health, Boston, MA USA; 5 Massachusetts Veterans Epidemiology Research and Information Center, VA Boston Healthcare System, Boston, MA USA; 6 Department of Epidemiology, Boston University School of Public Health, Boston, MA USA; 7 Department of Psychiatry, Boston University School of Medicine, Boston, MA USA; 8 Military Performance Division, US Army Research Institute of Environmental Medicine, Natick, MA USA

**Keywords:** Gulf War, Veterans, Toxic wounds, Neurotoxicant exposure, Longitudinal Design, Health symptoms

## Abstract

**Background:**

Thirty years ago, Gulf War (GW) veterans returned home with numerous health symptoms that have been associated with neurotoxicant exposures experienced during deployment. The health effects from these exposures have been termed toxic wounds. Most GW exposure-outcome studies utilize group analyses and thus individual fluctuations in symptoms may have been masked. This study investigates health symptom trajectories in the same veterans over 25 years.

**Methods:**

Veterans were categorized into 5 a priori trajectory groups for each health symptom and Chronic Multisymptom Illness (CMI) clinical case status. Multinomial logistic regression models were used to investigate associations between these trajectories and neurotoxicant exposures.

**Results:**

Results indicate that more than 21 Pyridostigmine Bromide (PB) pill exposure was associated with consistent reporting of fatigue, pain, and cognitive/mood symptoms as well as the development of six additional symptoms over time. Chemical weapons exposure was associated with both consistent reporting and development of neurological symptoms over time. Reported exposure to tent heater exhaust was associated with later development of gastrointestinal and pulmonary symptoms. Veterans reporting exposure to more than 21 PB pills were more than 8 times as likely to consistently meet the criteria for CMI over time.

**Conclusion:**

This study highlights the importance of the continued documentation of the health impacts experienced by GW veterans’, their resulting chronic health symptoms, and the importance of exposure-outcome relationships in these veterans now 30 years post-deployment.

## Background

Thirty years ago, Gulf War (GW) veterans returned home from deployment with a constellation of health symptoms: some have been chronic, some have emerged over time, and others have remitted [1, 2]. Collectively, these symptoms are termed Gulf War Illness (GWI) and have been associated with central nervous system (CNS) dysfunction as a result of neurotoxicant exposures during the war and resultant toxic wounds [3, 4]. These exposures include chemical warfare agents (sarin/cyclosarin), pesticide sprays and creams, pyridostigmine bromide (PB) prophylactic anti-nerve gas pills, smoke from oil well fires, tent heater exhausts, and others [3–6].

In the early years, research focused on determining the symptoms that characterized GWI, and identifying potential neurotoxicant exposures that could be etiologically related to these symptoms. However, studies which compared GWI cohorts to controls did not allow for the comparison of the separate GWI health symptom trajectories of an individual veteran. The majority of the studies employ the two most widely used case criteria for GWI, the Center for Disease Control (CDC)‘s Chronic Multisymptom Illness (CMI) criteria and the Kansas GWI criteria [7, 8]. To date, these two criteria are recommended by the Institute of Medicine (IOM) and Department of Defense (DoD) for use in GWI research studies. However, both criteria were derived over 20 years ago and reflect what the illness looked like initially post-deployment. In addition, these criteria ask veterans whether they have experienced these symptoms over the past 6 months. Therefore, the case criteria only capture current symptoms and do not account for potential changes in symptoms over time [9]. This has made obtaining service-related benefits difficult for veterans with toxic wounds.

Specifically, the CDC criteria include the following symptom domains: fatigue, mood and cognition, and pain, whereas the Kansas criteria also include gastrointestinal, respiratory, and skin domains [7, 8]. Additionally, the Kansas criteria exclude concomitant illnesses that could account for their chronic health symptoms, including neurologic disorders such as Parkinson’s disease, dementia, and stroke [8]. Relying on these case definitions 30 years post-deployment, researchers and clinicians may not be adequately addressing the sensitivity and specificity of the illness as a whole, as some symptoms may have decreased and new symptoms may have emerged over time [1, 10].

Importantly, these two criteria vary greatly in the presumptive rates of illness. The CDC criteria is likely to have increased rates of cases among various cohorts of GW veterans relative to the Kansas criteria (i.e. higher sensitivity but lower specificity) because the Kansas criteria encompasses more bodily systems and excludes those with other neurologic or other chronic medical conditions [7, 8]. For example, the Kansas criteria excludes diabetes and stroke, when studies have shown that these conditions are increasing in GW veterans over time and are related to specific neurotoxicant exposures (PB and sarin exposure) [2, 10]. These exposures may also be related to delayed or latent health symptoms that develop over time and can result in additional toxic wounds [1]. These strict Kansas criteria exclusions may have helped to characterize the GW veteran population initially, but now, 30 years later, may not be as applicable and may be excluding the very veterans who are the sickest and most affected by their deployment. For instance, in our recent study utilizing the population-based Ft. Devens Cohort (FDC) of GW veterans, we found that rates of both the CDC and Kansas case criteria have increased substantially (by 20%), over the course of 20 years in these largely non-treatment seeking veterans [2]. However, rates of Kansas GWI criteria continue to be lower than the CDC’s CMI criteria when those with chronic medical conditions are excluded (rates of cases at 66 and 79% respectively) [2].

An additional criticism of these criteria is that some of the symptoms may be sensitive to changes that are associated with normal aging (i.e., joint pain and sleep dysfunction), and thus may not reflect the actual deployment-related illness [11]. In a recent paper using the Department of Veteran Affairs’ Millennium Cohort, rates of CMI were compared between GW-era and non-era veterans over five separate time periods [12]. It was found that rates of CMI increased in all veteran groups over time, especially in those deployed to the Gulf region. The increased rates of CMI over time in other potentially non-exposed cohorts may suggest that the symptoms used in this criterion may be susceptible to non-deployment related factors, including normal aging. Further, as the GW veteran population ages and the initial rule-out diagnoses of the Kansas criteria are increasing in prevalence, it is imperative that we assess and potentially revise the discrete symptoms and exclusionary criteria. Reevaluating the diagnostic criteria will lessen the risk of excluding those individuals whose symptoms and medical conditions are related to GW deployment and who also have neurotoxicant-induced accelerated age-related disorders. Additionally, a revised criteria should exclude those who might erroneously meet criteria due to mild symptoms that are expected for their current age-group (i.e., joint paint and sleep dysfunction that increased with age) [10].

A recent re-survey (2014–2016) of the Gulf War Era Cohort and Biorepository (GWECB) was conducted to assess the current rates of GWI using both the CDC and the Kansas criteria, and to evaluate the utility of these criteria now to distinguish health problems reported by GW deployed veterans and GW era veterans [13]. The results of this study replicate what was found in Porter, et al [12], in that 80% of non-deployed era veterans reported symptoms consistent with the CDC GWI criteria, suggesting that this criteria has reduced specificity [13]. For the Kansas criteria, deployment status was associated with increased odds of reporting 27 of the 29 symptoms, suggesting its continued utility [13]. However, roughly 40% of the GWECB cohort had at least one medical or psychiatric condition that is considered an exclusion condition in the Kansas criteria. This is a more than 30% increase from the original Kansas cohort, further emphasizing the fact that these exclusion criteria need to be re-evaluated as GW veterans continue to age [13].

One possible explanation for variability of case status and corresponding health symptoms across samples reported in the literature is that different exposures may result in different types, rates, and longevity and trajectory of health symptoms [9, 14, 15]. Therefore, it is vitally important to continue to characterize the illness by toxicant exposures, as some toxicant exposures are known to exert latent or delayed health effects, for instance, chronic respiratory problems and cancers including brain tumors [16–19]. Although exposure outcome studies have been conducted with both preclinical and clinical models, most are conducted with exposure mixtures or by case status (GWI versus controls), so it is often difficult to conclude which symptoms result from specific exposures. Additionally, exposure-outcome relationship studies are often flawed because of the small sample sizes, limited exposure data, reliance on self-report or retrospective recall of exposure, cross-sectional analyses, or the use of treatment seeking populations with numerous health complaints [3, 4].

It is especially important to conduct exposure-outcome studies longitudinally in population-based non-treatment seeking cohorts. To date, several longitudinal studies of health symptoms and rates of case criteria have been conducted in GW veterans from multiple countries including the US, UK and Australia [1, 2, 9, 12, 20–22]. However, few studies have analyzed exposure-based outcomes longitudinally [1, 12]. We recently reported on data from the longest-running population-based cohort of GW veterans who returned from the war through Ft. Devens, MA (FDC) where we examined exposures related to health symptom outcomes [1]. We found increased rates of mood and cognitive outcomes in those reporting exposures to tent heaters, PB pills, and chemical warfare agents, and that the self-reported rates of these symptoms increased over time [1]. However, the analyses for this paper examined changes at the group rather than the individual level. Thus, it was not clear if specific symptoms fluctuated over time within individual veterans. For example, while the study showed that many symptoms increased over time, whether individual GW veteran symptom reporting increased, decreased, or fluctuated over time was not examined.

Assessing individual veteran health symptom trajectories is also important when analyzing trends in rates of a chronic disorder such as GWI. For example, there are many ways in which an individual could meet criteria for the illness. In analyses investigating rates of illness longitudinally, it is often unclear if the individuals are arriving at clinical status via the same symptoms, or potentially if some of their symptoms have recovered or if new ones have emerged, thus having the veteran continue to meet GWI criteria but through different combinations of symptoms. For example, there are 27 different ways in which an individual can meet the CDC’s CMI criteria [23]. Through group analyses, this may indicate that individuals have consistently met the criteria over time, when in fact, various symptoms may have fluctuated or appeared over time, but the individual’s case status remained unchanged (i.e. they still met criteria but by a different combination of symptoms).

Therefore, the most accurate way of assessing changing symptoms over time is to evaluate health symptom reporting longitudinally, in a way that assesses individual fluctuations in reporting, that may be masked in group analyses. To do this, each individual can be assigned into a trajectory group for every health symptom (i.e., no symptom, develops symptom, mixed reporting of the symptom, remission of symptom, and consistent report of symptom). The FDC, a cohort of deployed GW veterans who have been followed since post-deployment in 1991 and at three time points since (1992–1993, 1997–1998, and 2013–2017), is one of the few cohorts which allows for this type of analysis. In this paper we evaluated the same 15 health symptoms as well as clinical case status (GWI) in three separate time periods over 25 years, comparing individual veterans’ fluctuation in symptoms, or ‘symptom trajectories’, over time. In addition, given the exposure data compiled from this cohort over the years (beginning almost immediately post-deployment before some exposures were identified as potentially causative), we analyzed different trajectories of symptoms and CMI clinical case status in association with self-reported neurotoxicant exposures in the GW theatre.

## Methods

### Participants and surveys

**Fig. 1:**
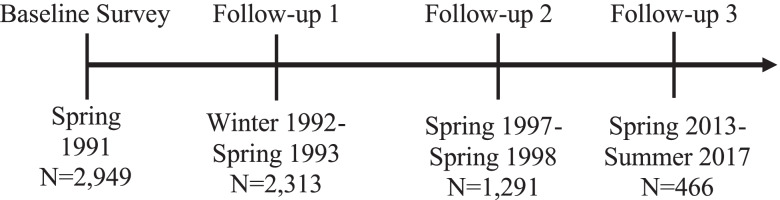
Ft. Devens cohort reunion survey timeline

The FDC has been described previously [1, 2]. Briefly, this was a cohort of Active Duty, Reserve, and National Guard Army veterans who were deployed to the GW and returned home through Ft. Devens, MA. The initial baseline survey in 1991 was designed to assess psychological health and combat exposure. Subsequent follow-up questionnaires (1992, 1997–1998, and 2013–2017) assessed long term health, psychological and functional well-being, including Post-traumatic stress disorder as measured by the Mississippi Scale for Combat Related PTSD ( [24] as well as self-report of GW specific neurotoxicant exposures. The current study uses a subset of the FDC participants (*N* = 293) who completed the Health Symptom Checklist at all three follow-up time points (1992, 1997–1998, and 2013–2017). All participants gave their informed consent for inclusion at each timepoint, before they participated in the surveys. Institutional review board approvals were obtained from VA Boston Healthcare System and Boston University prior to initiating the surveys. The timeline of the FDC is displayed in Fig. 1.

### The health symptom checklist

The minor differences in Health Symptom Checklist (HSC) versions across the three follow-up surveys have been explained in a previous study [1]. In brief, the HSC is a list of health symptoms originally adapted from Bartone, Ursano [25]. Follow-up 1 contained a 20-item HSC, in which veterans were asked to indicate the frequency of symptoms over the past 4 weeks using a Likert-scale rating (none, a little, often, very often) [26], Follow-up 2 contained a 52-item HSC, in which veterans were asked to indicate whether they experienced each health symptom over the past 4 weeks (yes or no) [27, 28], and Follow-up 3 contained a 34-item HSC, in which veterans were asked to endorse whether they experienced a symptom (yes or no) over the past 30 days [1]. A total of 15 symptoms were consistently assessed at all three timepoints. All responses were dichotomized as present or absent. If a veteran indicated not experiencing a symptom, and also endorsed a frequency, the response was recoded as being present.

### CMI criteria

Chronic Multisymptom Illness (CMI) by the Center for Disease Control (CDC) criteria were determined as the presence of persistent symptoms over six months in two out of three domains: fatigue, cognitive/mood, and musculoskeletal [7]. Because a limited number of symptoms [15] were consistently assessed at all three timepoints, our measure of the CMI criterion included: 1 symptom in the fatigue domain (overly tired/lack of energy), 4 in the cognitive/mood domain (depressed mood, difficulty concentrating, nervous or tense, trouble sleeping), and 1 in the musculoskeletal domain (joint pain).

### Gulf War exposure characterization

The full explanation of how GW exposures were characterized in the FDC has been reported previously [1]. In brief, to minimize the length of time between deployment and recall, exposure data were taken from the Follow-up 2 survey, 6 years after deployment. In this paper we report the associations between health symptom trajectories and 4 exposures, including PB pills, chemical weapon alert, Khamisiyah weapons depot notification, and tent heater exposure.

Participants were asked to indicate how many anti-nerve gas (PB) pills they took in the GW, on a scale of 0, 1–2, 3–10, 11–21, and > 21. Responses were coded into whether they took more than 21 PB pills, dichotomized into yes (> 21) and no (< 21) responses, as the blister pack given troops included 21 pills and 21 pills has previously been associated with higher rates of GWI symptoms [29]. Similarly, participants were asked to indicate how many times they were on “formal alert” for a chemical attack (e.g., had to put on full MOPP gear) on a scale of 0, 1–2, 3–10, 11–20, and 20 or more times. Responses were coded into whether they were on “formal alert” for more than 20 times, dichotomized into yes (> 20) and no (< 20) responses, as self-reported frequency of chemical alarms has been associated with adverse effects on brain structure, with the strongest effect observed for both 7–30 days and 30+ days of hearing chemical alarms [30].

Veteran’s exposure to the Khamisiyah weapons demolition was determined. In March of 1991, the Army Corp of engineers detonated underground munition bunkers which unbeknownst to them housed thousands of Iraqi rockets, with chemical weapons sarin/cyclosarin in the tips of the rockets. When the explosions occurred, it was estimated that over 100,000 GW veterans were exposed to low-level sarin/cyclosarin due to their proximity to the air plumes from the explosions [31, 32]. The Department of Defense (DoD) eventually notified exposed veterans by letter and a registry was established. A list of FDC veterans who received notification letters of potential exposure to sarin based on their proximity to the Khamisiyah weapons depot detonations and presumed to be exposed to low levels of sarin/cyclosarin based on wind plume modeling in 2000 was obtained from the DoD. These exposures were categorized as a dichotomous variable, yes (received a notification letter from the 2000 DOD exposure model) or no (never received a notification letter) for Khamisiyah exposure status as a proxy for sarin chemical weapons exposure during their deployment [32].

Lastly, participants were also asked whether they had a tent heater or stove in the area where they slept (yes vs. no).

### Symptom (Sx) trajectory groups

In order to evaluate health symptom trajectories over time, five a priori symptom trajectory groups were created based on responses at the three time points (No Sx, No Sx then Develops Sx, Mixed, Remitting Sx, Consistent Sx) for each symptom assessed (see Table 1). The No Sx group contains veterans who never endorsed the symptom on all three follow-up surveys. The No Sx then Develops Sx group contains both veterans who initially did not report the symptom on the first follow-up survey, but later endorsed the symptom on Follow-up 2 and Follow-up 3 or veterans who initially did not report the symptom on the first two follow-up surveys but endorsed on Follow-up 3. The Mixed group contains veterans whose symptoms of endorsement fluctuated across the follow-ups (i.e., endorsed on follow-up 1, not endorsed on follow-up 2, and then endorsed again on follow-up 3; and not endorsed on follow-up 1 endorse on follow-up 2 and then not endorsed again on follow-up 3). The Remitting Sx group contains veterans who initially endorsed the symptom on follow-up 1 but they did not endorse the symptom on follow-up 2 and 3, as well as veterans who initially endorsed the symptom on follow-ups 1 and 2 but did not endorse on follow-up 3. Finally, the Consistent Sx group contains veterans who endorsed the symptom on all three follow-up surveys. Participants were classified into trajectory groups for each health symptom and CMI clinical case status.

**Table 1:** Descriptions of Symptom Trajectory Groups

Symptom Trajectory Group	Follow-up 1	Follow-up 2	Follow-up 3
No Sx	N	N	N
No Sx, Develops Sx	N	N	Y
	N	Y	Y
Mixed	N	Y	N
	Y	N	Y
Remitting Sx	Y	Y	N
	Y	N	N
Consistent Sx	Y	Y	Y

Y = Endorsed Symptom N=Did Not Endorse Symptom

### Data analysis

Demographics and characteristics of the full FDC and the current study sample were presented using descriptive statistics. A multinomial logistic regression model was built with each health symptom trajectory grouping as the dependent variable, and one of the GW-specific exposures (i.e., PB pills, self-reported exposure to chemical warfare agents, notification of proximity to Khamisiyah sarin/cyclosarin air plumes, and tent heater exposure) as the independent variable. Covariates included in the model were age, sex, and post-traumatic stress disorder (PTSD) status (score of > 89 on the Mississippi PTSD Scale indicative of PTSD), all derived from the baseline survey conducted in 1991. We chose to include baseline PTSD status as a covariate to control for the cognitive, mood, and somatic symptoms that may arise from trauma, and therefore not neurotoxicant exposures [33, 34] . Additionally, for each individual exposure model, we included all other neurotoxicant exposures as covariates, to control for effects on symptoms from other exposures. Odds ratios (OR) and 95% confidence intervals (CI) were calculated from the logistic regression models. *P* values < 0.05 were considered significant. All analyses were performed using SAS 9.4 (SAS, Cary, NC).

## Results

### Demographics and baseline characteristics

A total of 2949 FDC veterans completed the initial baseline survey in 1991. A total of 295 veterans completed all follow-up surveys. Two veterans did not have complete health symptom data for all three follow-up surveys and were thus excluded from the study sample for a final sample size of 293. The veterans in the study sample were approximately 32 years old at the time of the initial 1991 baseline survey and 54 years old by the last follow-up survey (Table 2). Veterans were predominately male (87.4%) and White (92.8%). Over 16% of the veterans were on active duty at the time of the GW and 5.5% of the study sample exceeded the clinical cutoff of the Mississippi scale for PTSD. The full cohort from the initial 1991 baseline survey did not differ from the study sample with regards to PTSD score or status. The study sample differed from the full FDC, as veterans were older, more likely to be White, and less likely to be male and initially on active duty. Demographics and characteristics for both the full FDC sample and the study sample are summarized in Table 2.

**Table 2:** Demographics and Characteristics

Demographics/Characteristics	Full Devens Cohort (*N* = 2949)	Study Sample (*N* = 293)
Age at baseline survey, years*	30.2 + 8.3	32.3 + 8.5
Age at follow-up 3, years		54.28 + 8.56
Male, n (%)*	2702 (91.6)	256 (87.4)
White, n (%)*	2443 (82.8)	272 (92.8)
Active Duty at time of Gulf War, n (%)* (versus Reserve, National Guard)	823 (27.9)	48 (16.4)
Mississippi PTSD scale-score	61.9 + 13.4	62.0 + 14.2
Clinical cutoff on Mississippi scale-score, n (%)	116 (3.9)	16 (5.5)
GW-Specific Neurotoxicant Exposures, n (%) Time 4 (*N* = 1291)		
Took more than 21 Pyridostigmine Bromide (PB) Pills	210 (16.3)	50 (17.1)
20 or More Times on “Formal Alert” for a Chemical Attack	263 (20.4)	70 (23.9)
Received the 2000 DoD Notification for Possible Sarin Exposure from the Khamisiyah Weapons Demolition*	1024 (34.7)	121 (41.3)
Tent Heater or Stove in the Area Where you Slept*	788 (61.0)	200 (68.3)

*p < 0.05

### Individual symptom trajectories

**Fig. 2:**
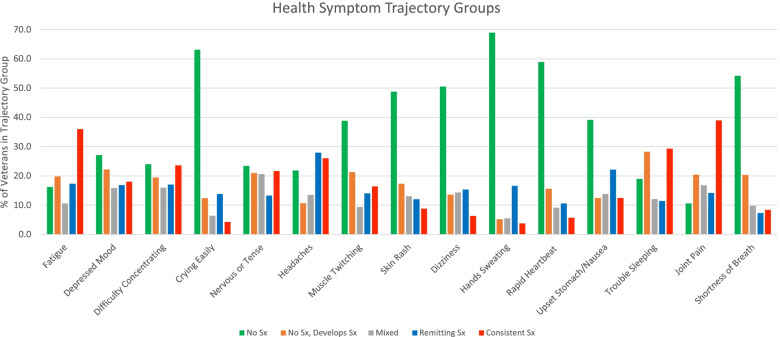
Percentage of veterans by symptom trajectory group for each health symptom

The prevalence of trajectory groups for each symptom are described in Fig. 2 (sample sizes of each group are presented in Table 3). Less commonly reported symptoms (greater than 50% of veterans never endorsed across all three follow-up surveys) were crying easily, hands sweating, rapid heartbeat, dizziness, and shortness of breath. Symptoms that showed an increase in prevalence over time (greater than 20% of veterans did not report initially but reported at later follow-ups) were muscle twitching, depressed mood, trouble sleeping, fatigue, nervousness, and joint pain. The only symptom that waxed and waned over time was nervous or tense (with 20% of veterans in the mixed trajectory group). Remitting symptoms included headaches and nausea (with more than 20% of veterans in these trajectory groups). More commonly reported symptoms (greater than 30% of veterans consistently reported these symptoms across all three follow-up surveys) were fatigue, trouble sleeping, and joint pain.

**Table 3:** Associations between GW-Specific Neurotoxicant Exposures and Symptom Trajectories Adjusted for other exposures

Outcome	Comparison	Khamisiyah OR [95% CI]	Tent Heater OR [95% CI]	PB Pill (21 or More) OR [95% CI]	Chemical Alert (20 or More) OR [95% CI]
Difficulty Concentrating	No Sx (*n* = 69)	Ref	Ref	Ref	Ref
	No Sx, Develops Sx (*n* = 56)	1.44 [0.57–3.66]	0.65 [0.26–1.61]	**3.53 [1.08–11.50]**	0.74 [0.26–2.15]
	Mixed (*n* = 46)	2.50 [0.95–6.58]	0.79 [0.29–2.14]	3.70 [1.00–13.70]	0.61 [0.19–2.00]
	Remitting Sx (*n* = 49)	1.87 [0.76–4.57]	0.73 [0.29–1.85]	1.56 [0.45–5.40]	1.33 [0.50–3.52]
	Consistent Sx (*n* = 68)	**3.09 [1.26–57.61]**	0.69 [0.27–1.75]	2.71 [0.81–9.13]	0.95 [0.34–2.63]
Dizziness	No Sx (*n* = 133)	Ref	Ref	Ref	Ref
	No Sx, Develops Sx (*n* = 39)	0.76 [0.30–1.93]	2.22 [0.83–5.91]	**4.87 [1.78–13.37]**	1.90 [0.70–5.17]
	Mixed (*n* = 37)	0.82 [0.32–2.07]	1.34 [0.53–3.42]	0.35 [0.07–1.84]	2.38 [0.86–6.60]
	Remitting Sx (*n* = 43)	1.09 [0.46–2.57]	1.31 [0.54–3.16]	2.33 [0.82–6.67]	1.54 [0.57–4.13]
	Consistent Sx (*n* = 18)	**6.49 [1.51–27.90]**	1.11 [0.27–4.49]	**8.59 [2.07–35.59]**	0.85 [0.19–3.85]
Fatigue	No Sx (n = 46)	Ref	Ref	Ref	Ref
	No Sx, Develops Sx (n = 56)	0.78 [0.28–2.16]	1.16 [0.44–3.08]	3.93 [0.98–15.78]	1.31 [0.44–3.94]
	Mixed (*n* = 30)	1.42 [0.46–4.39]	1.35 [0.44–4.17]	4.06 [0.85–19.52]	0.67 [0.16–2.70]
	Remitting Sx (n = 49)	1.28 [0.48–3.42]	**3.02 [1.05–8.68]**	1.80 [0.39–8.33]	0.69 [0.21–2.31]
	Consistent Sx (*n* = 101)	1.30 [0.53–3.19]	1.47 [0.60–3.61]	**3.76 [1.02–13.88]**	1.03 [0.37–2.88]
Crying Easily	No Sx (*n* = 178)	Ref	Ref	Ref	Ref
	No Sx, Develops Sx (*n* = 35)	1.02 [0.38–2.70]	2.23 [0.76–6.57]	1.73 [0.56–5.36]	1.15 [0.37–3.57]
	Mixed (n = 18)	0.85 [0.21–3.43]	3.43 [0.62–19.01]	1.67 [0.32–8.68]	1.29 [0.27–6.13]
	Remitting Sx (n = 39)	0.79 [0.29–2.16]	**4.12 [1.18–14.39]**	0.88 [0.26–3.04]	1.90 [0.66–5.47]
	Consistent Sx (n = 12)	0.81 [0.15–4.28]	Sample Size Issue	2.66 [0.43–15.52]	2.02 [0.32–12.65]
Nervous or Tense	No Sx (*n* = 67)	Ref	Ref	Ref	Ref
	No Sx, Develops Sx (*n* = 60)	1.12 [0.47–2.65]	0.99 [0.43–2.29]	**3.80 [1.03–14.05]**	1.21 [0.44–3.38]
	Mixed (*n* = 59)	1.42 [0.58–3.45]	1.91 [0.75–4.82]	**3.99 [1.05–14.99]**	1.06 [0.36–3.13]
	Remitting Sx (*n* = 38)	0.88 [0.33–2.32]	2.16 [0.80–5.88]	2.87 [0.68–12.03]	1.48 [0.49–4.45]
	Consistent Sx (*n* = 62)	1.14 [0.44–2.99]	1.78 [0.65–4.84]	**7.42 [1.89–29.09]**	0.97 [0.30–3.08]
Upset Stomach/Nausea	No Sx (*n* = 113)	Ref	Ref	Ref	Ref
	No Sx, Develops Sx (*n* = 36)	0.35 [0.12–1.01]	**5.42 [1.45–20.29]**	0.64 [0.15–2.69]	0.57 [0.14–2.26]
	Mixed (*n* = 40)	**0.33 [0.11–0.93]**	0.96 [0.37–2.49]	0.60 [0.15–2.44]	1.74 [0.62–4.92]
	Remitting Sx (*n* = 64)	**0.37 [0.16–0.82]**	1.31 [0.60–2.90]	1.40 [0.55–3.53]	0.88 [0.36–2.13]
	Consistent Sx (n = 36)	0.85 [0.30–2.38]	2.68 [0.79–9.13]	**3.27 [1.03–10.31]**	1.02 [0.32–3.28]
Shortness of Breath	No Sx (*n* = 155)	Ref	Ref	Ref	Ref
	No Sx, Develops Sx (*n* = 58)	0.76 [0.34–1.72]	**3.46 [1.34–8.95]**	**2.68 [1.07–6.70]**	1.61 [0.66–3.92]
	Mixed (*n* = 28)	1.00 [0.29–3.38]	2.02 [0.51–8.02]	2.39 [0.60–9.54]	1.39 [0.36–5.42]
	Remitting Sx (*n* = 21)	1.27 [0.43–3.72]	1.74 [0.51–5.94]	2.07 [0.55–7.78]	0.57 [0.14–2.44]
	Consistent Sx (*n* = 24)	2.61 [0.74–9.24]	**0.22 [0.05–0.88]**	2.15 [0.43–10.70]	1.51 [0.38–6.02]
Depressed Mood	No Sx (*n* = 77)	Ref	Ref	Ref	Ref
	No Sx, Develops Sx (*n* = 63)	2.36 [0.99–5.63]	0.93 [0.40–2.19]	**4.46 [1.34–14.80]**	0.91 [0.33–2.47]
	Mixed (*n* = 45)	1.51 [0.56–4.09]	0.83 [0.32–2.15]	**3.90 [1.09–13.98]**	1.18 [0.41–3.46]
	Remitting Sx (*n* = 48)	**3.19 [1.32–7.73]**	1.23 [0.49–3.08]	3.10 [0.88–10.94]	1.06 [0.39–2.93]
	Consistent Sx (*n* = 51)	**2.99 [1.08–8.31]**	1.12 [0.39–3.23]	**7.01 [1.82–26.99]**	1.00 [0.31–3.24]
Headaches	No Sx (n = 63)	Ref	Ref	Ref	Ref
	No Sx, Develops Sx (*n* = 31)	0.34 [0.11–1.04]	0.87 [0.30–2.50]	0.69 [0.15–3.28]	1.15 [0.30–4.48]
	Mixed (n = 39)	1.00 [0.36–2.78]	0.94 [0.32–2.72]	0.65 [0.13–3.22]	**4.10 [1.29–13.04]**
	Remitting Sx (*n* = 81)	**0.34 [0.15–0.78]**	1.54 [0.65–3.62]	2.10 [0.72–6.14]	1.00 [0.35–2.91]
	Consistent Sx (*n* = 75)	0.44 [0.18–1.07]	0.76 [0.31–1.86]	2.82 [0.91–8.75]	2.09 [0.73–6.00]
Muscle Twitching	No Sx (*n* = 111)	Ref	Ref	Ref	Ref
	No Sx, Develops Sx (*n* = 61)	1.03 [0.47–2.24]	1.26 [0.58–2.74]	1.51 [0.57–4.00]	1.26 [0.50–3.17]
	Mixed (*n* = 27)	0.87 [0.30–2.50]	0.80 [0.29–2.23]	1.49 [0.41–5.50]	0.83 [0.23–3.02]
	Remitting Sx (n = 40)	0.94 [0.38–2.27]	2.39 [0.90–6.36]	0.96 [0.30–3.08]	1.39 [0.50–3.92]
	Consistent Sx (*n* = 47)	0.98 [0.39–2.48]	**3.11 [1.07–9.06]**	2.18 [0.74–6.43]	2.23 [0.81–6.15]
	No Sx, (*n* = 138)	Ref	Ref	Ref	Ref
	No Sx, Develops Sx (n = 49)	0.55 [0.22–1.39]	**0.40 [0.17–0.94]**	**4.72 [1.63–13.71]**	**0.17 [0.04–0.67]**
	Mixed (n = 37)	1.60 [0.61–4.23]	1.22 [0.40–3.70]	1.83 [0.58–5.82]	2.31 [0.83–6.45]
	Remitting Sx (*n* = 34)	0.77 [0.30–2.01]	1.23 [0.43–3.51]	1.64 [0.46–5.75]	0.73 [0.23–2.29]
	Consistent Sx (*n* = 25)	1.27 [0.41–3.91]	0.62 [0.19–2.00]	1.78 [0.43–7.37]	2.00 [0.60–6.62]
Hands Sweating	No Sx (*n* = 199)	Ref	Ref	Ref	Ref
	No Sx, Develops Sx (n = 15)	0.53 [0.10–2.68]	2.53 [0.50–12.75]	3.16 [0.73–13.66]	1.43 [0.32–6.37]
	Mixed (*n* = 16)	**4.09 [1.03–16.22]**	2.43 [0.47–12.56]	**4.47 [1.06–18.81]**	1.53 [0.36–6.52]
	Remitting Sx (n = 48)	1.43 [0.60–3.40]	1.51 [0.59–3.86]	2.32 [0.86–6.26]	1.23 [0.47–3.21]
	Consistent Sx (n = 11)	5.22 [0.44–61.56]	2.36 [0.15–38.03]	3.18 [0.29–35.23]	1.65 [0.17–15.81]
Trouble Sleeping	No Sx (*n* = 55)	Ref	Ref	Ref	Ref
	No Sx, Develops Sx (*n* = 82)	0.72 [0.31–1.69]	1.35 [0.57–3.18]	1.43 [0.40–5.12]	1.12 [0.42–3.03]
	Mixed (n = 35)	0.79 [0.27–2.27]	0.82 [0.29–2.32]	3.30 [0.82–13.26]	1.47 [0.46–4.71]
	Remitting Sx (*n* = 33)	1.09 [0.39–3.05]	1.04 [0.37–2.97]	2.57 [0.63–10.42]	0.67 [0.18–2.41]
Rapid Heartbeat	No Sx (*n* = 166)	Ref.	Ref.	Ref.	Ref.
	No Sx, Develops Sx (*n* = 44)	0.89 [0.39–2.05]	1.19 [0.52–2.74]	1.53 (0.57–4.14]	1.33 [0.54–3.27]
	Mixed (*n* = 26)	1.36 [0.46–4.06]	1.04 [0.34–3.15]	1.90 [0.51–7.11]	0.46 [0.10–2.03]
	Remitting Sx (n = 30)	1.19 [0.42–3.38]	2.64 [0.70–9.89]	1.95 [0.57–6.69]	0.64 [0.17–2.37]
	Consistent Sx (n = 16)	1.45 [0.42–5.01]	1.45 [0.38–5.59]	2.27 [0.54–9.51]	1.15 [0.29–4.54]
Joint Pain	No Sx (n = 29)	Ref	Ref	Ref	Ref
	No Sx, Develops Sx (n = 56)	1.16 [0.39–3.46]	1.04 [0.35–3.09]	Sample Size Issue	0.67 [0.20–2.25]
	Mixed (n = 46)	0.99 [0.31–3.15]	0.91 [0.30–2.79]	Sample Size Issue	0.34 [0.09–1.38]
	Remitting Sx (n = 39)	1.81 [0.58–5.68]	1.04 [0.33–3.33]	Sample Size Issue	0.64 [0.17–2.34]
	Consistent Sx (*n* = 104)	1.44 [0.50–4.15]	1.31 [0.45–3.81]	Sample Size Issue	0.84 [0.26–2.67]

All analyses included age, gender, and baseline PTSD status as covariates. ***p*** **< 0.05**

**Fig. 3:**
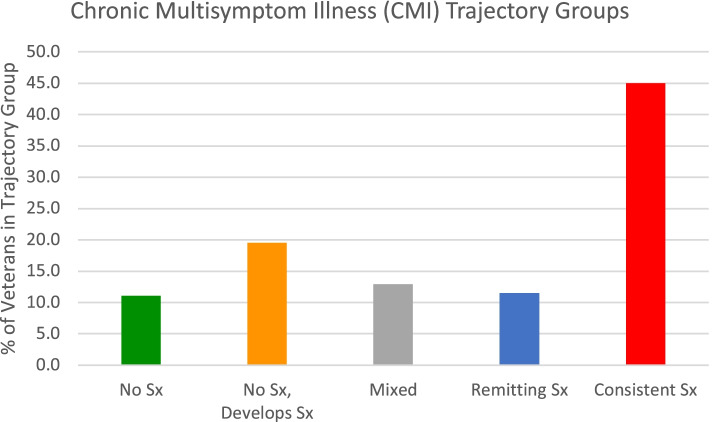
Percentage of veterans by chronic multisymptom illness (CMI) trajectory group

The prevalence of trajectory groups for CMI clinical case status are described in Fig. 3 (sample sizes of each group are presented in Table 4). Most veterans (45%) consistently met criteria for CMI across all three follow-ups, followed by 19.5% of veterans who met criteria for CMI as time went on, 12.9% of veterans had a mixed trajectory of CMI clinical case status, and 11.1% of veterans never met criteria for CMI across all follow-ups.

**Table 4:** Associations between Exposures and CMI – GWI Trajectories

Exposure	Outcome	Comparison	OR 95% CI
Khamisiyah	CMI - GWI	No Sx (*n* = 32)	Ref
		No Sx, Develops Sx (n = 56)	1.46 [0.47, 4.49]
		Mixed (n = 37)	1.56 [0.47, 5.20]
		Remitting Sx (n = 33)	3.26 [0.97, 10.89]
		Consistent Sx (*n* = 129)	1.58 [0.56, 4.43]
Tent Heater	CMI - GWI	No Sx (n = 32)	Ref
		No Sx, Develops Sx (n = 56)	0.82 [0.28, 2.36]
		Mixed (n = 37)	1.80 [0.54, 6.06]
		Remitting Sx (n = 33)	2.02 [0.57, 7.14]
		Consistent Sx (*n* = 129)	1.10 [0.41, 2.91]
PB Pills (21 or more)	CMI - GWI	No Sx (n = 32)	Ref
		No Sx, Develops Sx (n = 56)	8.65 [0.97, 76.96]
		Mixed (n = 37)	8.51 [0.90, 80.24]
		Remitting Sx (n = 33)	3.44 [0.31, 38.45]
		Consistent Sx (n = 129)	**8.75 [1.03, 74.16]**
Chemical Alert (20 or more)	CMI - GWI	No Sx (n = 32)	Ref
		No Sx, Develops Sx (n = 56)	1.44 [0.38, 5.38]
		Mixed (n = 37)	1.11 [0.26, 4.77]
		Remitting Sx (n = 33)	1.59 [0.38, 6.69]
		Consistent Sx (n = 129)	1.35 [0.39, 4.62]

All analyses included age, gender, and baseline PTSD status as covariates. ***p* < 0.05**

### Associations between individual symptom trajectories and neurotoxicant exposures

#### PB pill (more than 21) exposure

Veterans who reported taking more than 21 PB pills were more than twice as likely to develop difficulty concentrating, dizziness, nervous or tense, shortness of breath, depressed mood, and skin rash over time, as compared to those reporting < 21 PB pill exposure (0–21 pills) (*p*’s < 0.05). This exposure was also associated with fluctuating reporting of nervous or tense, hands sweating, and depressed mood over time (*p*’s < 0.05). No associations were observed between PB pill (more than 21) exposure and symptom trajectories of headaches, muscle twitching, rapid heartbeat and crying easily (*p*’s > 0.05). Veterans who reported taking more than 21 PB pills were more than twice as likely to consistently report these symptoms over three time periods, including the following: dizziness, fatigue, nervous or tense, trouble sleeping, upset stomach/nausea, and depressed mood as compared to those reporting < 21 PB pill exposure (0–21 pills) (*p*’s < 0.05). These results and odds ratios are reported in Table 3.

#### Chemical alert (20 or more) exposure

Veterans who reported experiencing 20 or more chemical alerts were more than four times as likely to fluctuate reporting of headaches over time (*p* < 0.05). Additionally, veterans who reported this exposure were *less* likely to develop skin rash over time (*p* < 0.05).

No associations were observed between chemical alert exposure (20 or more times) and symptom trajectories of hands sweating, trouble sleeping, rapid heartbeat, joint pain, difficulty concentrating, dizziness, fatigue, crying easily, nervous or tense, upset stomach/nausea, shortness of breath, depressed mood, and muscle twitching (*p*’s > 0.05). These results and odds ratios are reported in Table 3.

#### Khamisiyah weapons demolition sarin air plume notification letter received –

Veterans who reported receiving a Khamisiyah notification letter from DoD were more than twice as likely to consistently report difficulty concentrating, dizziness, and depressed mood over the three time periods assessed compared to those who did not report receiving a Khamisiyah notification letter (*p*’s < 0.05). Those reporting this exposure were also more than 3 times as likely to recover from depressed mood over time (*p* < 0.05). Those reporting this exposure were more than 4 times as likely to fluctuate on reporting hands sweating (*p* < 0.05). Those reporting this exposure were significantly less likely to fluctuate in reporting upset stomach/nausea or to recover from this symptom over time (*p*’s < 0.05). Those reporting this exposure were significantly less likely to report headaches initially and then recover over time (*p* < 0.05). Significant associations were not observed between Khamisiyah exposure and symptom trajectories of shortness of breath, muscle twitching, skin rash, trouble sleeping, rapid heartbeat, joint pain, fatigue, crying easily, and nervous or tense (*p*’s > 0.05). These results and odds ratios are reported in Table 3.

#### Tent heater exposure

Veterans who reported tent heater exposure were more than three times as likely to develop upset stomach/nausea and shortness of breath over time compared to those reporting no exposure to tent heaters (*p*’s < 0.05). Additionally, those reporting this exposure were less likely to develop skin rash over time and less likely to consistently report shortness of breath, compared to those reporting no exposure to tent heaters (*p* < 0.05). Those who reported this exposure were more than 3 times as likely to consistently report muscle twitching over time (*p* < 0.05). Veterans who reported this exposure were also three times as likely to recover from fatigue and crying easily over time as compared to those not reporting tent heater exposure (*p* < 0.05). No associations were observed between tent heater exposure and symptom trajectories of hands sweating, trouble sleeping, rapid heartbeat, joint pain, difficulty concentrating, dizziness, nervous or tense, depressed mood, and headaches (*p*’s > 0.05). These results and odds ratios are reported in Table 3.

#### Chronic multisymptom illness modified criteria (CMI)

**Fig. 4:**
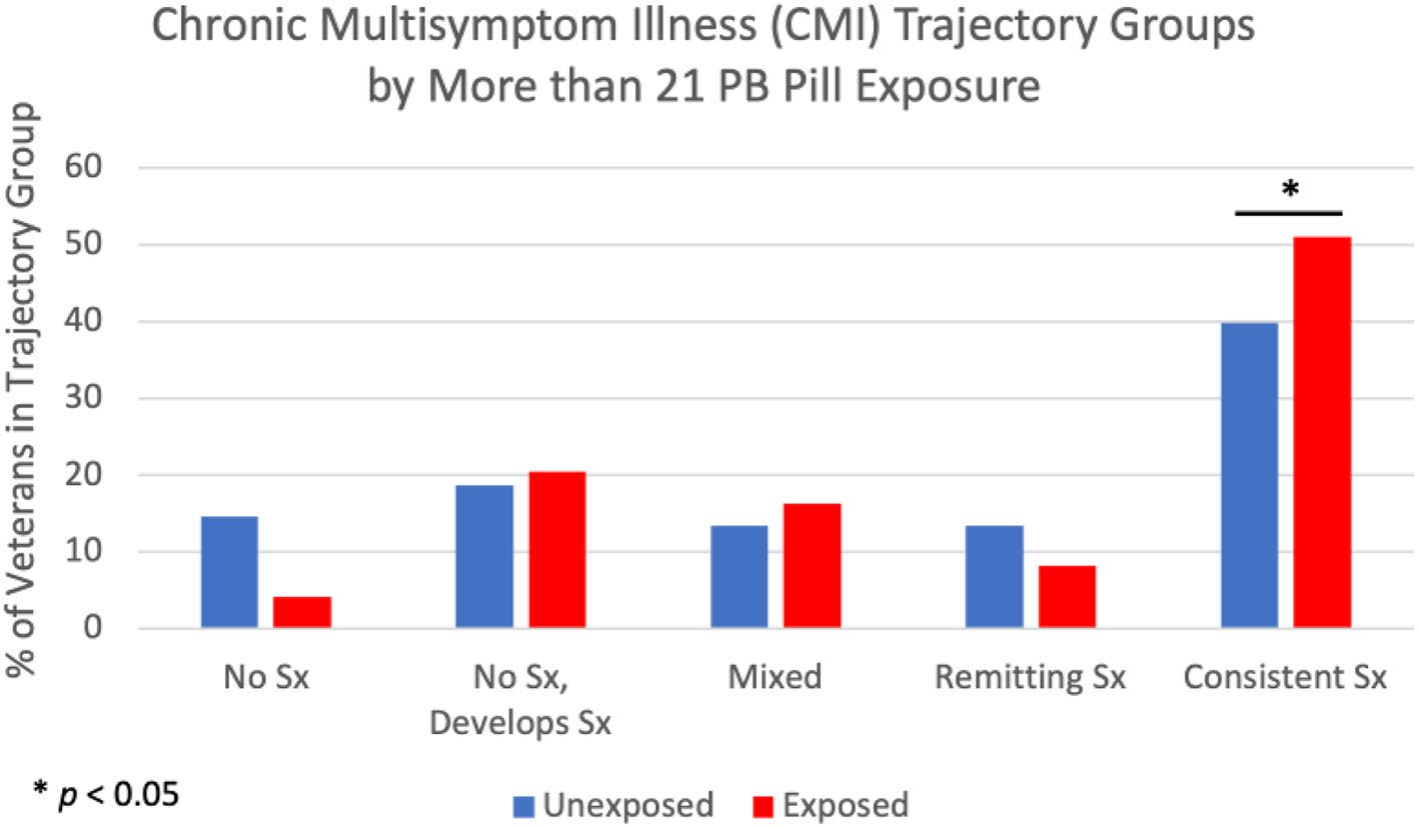
Percentage of veterans in chronic multisymptom illness (CMI) trajectory group by more than 21 pyridostigmine bromide (PB) pill exposure

Veterans who reported taking more than 21 PB pills were more than 8 times as likely to consistently meet the criteria for CMI over time compared to those reporting < 21 PB pill exposure (0–21 pills) (*p* < 0.05). No significant associations were observed for chemical alert exposure, Khamisiyah weapons demolition exposure, or tent heater exposure for trajectories of meeting the CMI criteria (*p*’s > 0.05). These results are summarized in Table 4 and Fig. 4.

## Discussion

To our knowledge, this study is the first of its kind to evaluate individual health symptom trajectories across three time periods over more than 25 years and associations with GW-specific neurotoxicant exposures. In addition, we do not know of another study where the same individuals were compared in terms of meeting case definition of CMI and exposures across three time-periods.

Our results showed that fatigue and general aches and pain/joint pain were identified as symptoms consistently endorsed by over 30% of this cohort of GW veterans at three time periods over the span of more than 25 years. These two symptoms are currently included in both the CDC and the Kansas criteria, and consist of two of the most prominent symptom domains: Fatigue and Pain [7, 8]. Four symptoms (headache, poor concentration, poor sleep, nervousness) were consistently reported by at least 20% of the cohort who were surveyed at three time points. The symptoms of poor concentration and nervousness are in the Kansas and CMI criteria of cognition and mood. Headache, nervousness, and poor sleep are also CNS symptoms that are associated with GW deployment that should be considered in future case criteria iterations [3]. These findings of consistent health concerns over 25 years in the same individuals also add credence for these symptoms’ future utility in biomarker and treatment development studies of GWI [2].

Only two symptoms, headaches (27%) and stomach aches/nausea (21%) were endorsed initially but not endorsed at later timepoints by a quarter and a fifth of the cohort respectively. We did not measure the extent to which targeted treatments were used for these symptoms, but it is possible that these individuals benefitted from such specialty clinic approaches at their local VA or private healthcare facilities.

Several symptoms were identified that were not apparent early on post-deployment but appeared later. These symptoms included fatigue, depression, nervousness, muscle aches, poor sleep, aches and pains, and shortness of breath, which were all endorsed by over 20% of the sample. Unique symptoms that were not reported above, and developed over time included mood, pain and respiratory domains, indicating their continued utility in current case criteria.

Several symptoms were never endorsed by at least 50% of our cohort. These included crying easily, hands sweating, skin rash, shortness of breath, and rapid heartbeat. These symptoms tend to represent autonomic nervous system (ANS) disturbance and although there have been reports of ANS symptoms in GW veterans, it may represent a smaller subgroup and may be consistent with particular toxicant exposure outcomes [35, 36]. Differences in symptom reporting were noted throughout the groupings, which may be related to the exposures that were experienced in theatre.

Several associations between GW-specific neurotoxicant exposures and health symptom trajectories were observed. PB exposure of more than 21 pills was associated with consistent reporting of fatigue and trouble sleeping, cognitive and mood symptoms, as well as nausea/upset stomach, and dizziness. Symptoms that developed over time in this exposed group included dizziness, difficulty concentrating, nervous/tense, shortness of breath, depressed mood, and skin rash. This exposure was not associated with any symptoms remitting over time. This exposure had associations with all symptom domains currently encompassed in the two case definitions for GWI [7, 8], suggesting a widespread effect of CNS-dysfunction affecting all major body systems, which is in line with previous studies that have found associations between this exposure and the illness (CMI) as a whole [7, 28]. They also represent many symptoms of the acetylcholinergic system which PB targets [37–39]. In fact, the current study found that veterans reporting PB exposure of more than 21 pills were more than eight times as likely to consistently meet the criteria for CMI over the span of more than 25 years, which is consistent with prior findings and expands upon the cross-sectional results of Steele, Sastre [40] and Wolfe, Proctor [28]. Over 250,000 were exposed to PB, which is a significant concern in considering a cause for the development of chronic health outcomes in these veterans [6, 38, 39]. These results suggest that an alternative to PB pills should be used in future deployments and that troops who were exposed to more than 21 PB pills should be considered under new legislative category of toxic wounds for service connection.

Self-reported exposure to 20 or more chemical alerts was associated with no consistent reporting of any of the symptoms. There were also no symptoms that developed later. However those who reported exposure to 20 or more chemical alerts were likely to show fluctuating reporting of headaches over time.

Suspected sarin exposure at the Khamisiyah weapons demolition was associated with consistent reporting of difficulty concentrating and dizziness over time, and depressed mood similar to what has been reported in previous studies and further validating the chronic CNS effects of these exposures (1, 42). However, they were also three times as likely to recover from depressed mood over time.

Tent heater exposure was associated with consistent reporting of shortness of breath and also to report the development of nausea/upset stomach and shortness of breath over time,. This latter finding is suggestive of a possible delayed or latent effect of this exposure on the gastrointestinal and respiratory systems. Veterans with this exposure were three times as likely to recover from fatigue and crying easily over time.. These findings are similar to other studies that have reported associations between tent heater exposure and neurological and pulmonary symptoms [27, 28]. However, this is the first study to our knowledge to report an association between tent heaters with gastrointestinal symptoms.

### Limitations and strengths

The current analysis was limited to veterans who responded and had complete health symptom data for all three follow-up studies, which may limit the generalizability of our results, not only to the greater FDC, but to the entire GW veteran population. For example, our study sample was more likely to be male and White and less likely to have been active duty during the GW than was found in the total Devens cohort. Confirmatory studies should be conducted in more representative samples.

Second, no objective measures of GW-specific neurotoxicant exposures are available. Therefore, although we used self-report measures of these exposures, we minimized recall bias by using exposure data from Follow-up 2, which was less than 5 years after veterans returned from the GW and before many exposure-outcome reports had been published. Further, the first two follow-up surveys were conducted before the DoD notification letters of suspected sarin exposure at the Khamisiyah weapons demolition were sent. Thus, veteran’s reporting of symptoms may be less biased, as they may not have known that they were exposed to sarin and would not attribute their symptoms to that exposure. In addition, exposures were reduced to binomial yes/no variables with yes denoting a specified amount based on prior research. Unfortunately, we did not have the statistical power to break these categories down. Exposure categories were not mutually exclusive. We were also limited to the exposure questions previously asked and therefore, we are not able to look at other acetylcholinesterase inhibitors and the impact on health symptoms over time.

Third, the current study was limited to using 15 health symptoms variables that were reported at all 3 follow-up surveys. It would be of interest to explore not only other health symptoms but also neuropsychological performance and neuroimaging markers longitudinally, to track disease progression, and to potentially identify new or latent effects of exposure. Next, while no data on potential treatment use were collected for this cohort, we acknowledge that treatment use could have affected not only the symptoms that were observed to resolve over time, but other symptom trajectories as well. Lastly, results should be interpreted with caution as analyses were not adjusted for multiple comparisons.

## Conclusions

This study highlights the importance of the continued documentation of GW veterans’ health status by GW-specific neurotoxicant exposures, and the importance of exposure-outcome relationships in these veterans now 30 years post-deployment. Importantly, several symptoms were identified that have been consistently endorsed for nearly 25 years post-deployment, suggesting chronic effects of the GW-specific neurotoxicant exposures. Therefore, the current recommended case criteria by Fukuda and Steele remain relevant based on this longitudinal analysis and could be further refined and updated with regard to exclusionary criteria and symptom inclusionary criteria based on our reported exposure-outcome relationships. Additionally, while several treatments addressing neuroinflammatory and neuroimmune mechanisms are currently being investigated through clinical trials, targeting symptom alleviation would improve veteran’s quality of life until treatments for the illness as a whole become widely available.

## Data Availability

Analyses were performed using raw data that are only available within the US Department of Veterans Affairs firewall in a secure research environment. VA privacy and data security policies and regulatory constraints provide that only aggregate summary data may be removed from the VA for publication. The authors have provided detailed results of these analyses in the paper. These restrictions are in place in order to maintain patient privacy and confidentiality. Access to these data can be granted to persons who are not an employee of the VA; however, there is an official protocol that must be followed for doing so. The authors invite those wishing to access the raw data that were used for this analysis to contact Maxine Krengel (Maxine.Krengel@va.gov) to discuss the details of the VA data access approval process.

## Abbreviations

ANS: Autonomic Nervous System
CDC: Center for Disease Control
CI: Confidence Interval
CMI: Chronic Multisymptom Illness
CNS: Central Nervous System
DoD: Department of Defense
FDC: Ft. Devens Cohort
GW: Gulf War
GWI: Gulf War Illness
HSC: Health Symptom Checklist
IOM: Institute of Medicine
MA: Massachusetts
MOPP: Mission Oriented Protective Posture
NC: North Carolina
OR: Odds Ratio
PB: Pyridostigmine Bromide
PTSD: Post Traumatic Stress Disorder
SAS: Statistical Analysis System
Sx: Symptom
US: United States
UK: United Kingdom
VA: Veterans Affairs
